# Elevated plasma phospholipid n-3 docosapentaenoic acid concentrations during hibernation

**DOI:** 10.1371/journal.pone.0285782

**Published:** 2023-06-09

**Authors:** Birgitta Strandvik, Abdul Rashid Qureshi, Johanna Painer, Carolina Backman-Johansson, Martin Engvall, Ole Fröbert, Jonas Kindberg, Peter Stenvinkel, Sylvain Giroud

**Affiliations:** 1 Department of Biosciences and Nutrition, Karolinska Institutet NEO, Stockholm, Sweden; 2 Division of Renal Medicine, CLINTEC, Karolinska Institutet, Stockholm, Sweden; 3 Research Institute of Wildlife Ecology, Department of Interdisciplinary Life Sciences, University of Veterinary Medicine, Vienna, Austria; 4 Department of Molecular Medicine and Surgery, Karolinska Institutet, Stockholm, Sweden; 5 Department of Cardiology, Faculty of Health, Örebro University, Örebro, Sweden; 6 Department of Clinical Medicine, Aarhus University Health, Aarhus, Denmark; 7 Department of Clinical Pharmacology, Aarhus University Hospital, Aarhus, Denmark; 8 StenoDiabetes Center Aarhus, Aarhus University Hospital, Aarhus, Denmark; 9 Department of Wildlife, Fish and Environmental Studies, University of Agricultural Sciences, Umeå, Sweden; 10 Norwegian Institute for Nature Research, Trondheim, Norway; Tokai University, JAPAN

## Abstract

Factors for initiating hibernation are unknown, but the condition shares some metabolic similarities with consciousness/sleep, which has been associated with n-3 fatty acids in humans. We investigated plasma phospholipid fatty acid profiles during hibernation and summer in free-ranging brown bears (*Ursus arctos*) and in captive garden dormice (*Eliomys quercinus*) contrasting in their hibernation patterns. The dormice received three different dietary fatty acid concentrations of linoleic acid (LA) (19%, 36% and 53%), with correspondingly decreased alpha-linolenic acid (ALA) (32%, 17% and 1.4%). Saturated and monounsaturated fatty acids showed small differences between summer and hibernation in both species. The dormice diet influenced n-6 fatty acids and eicosapentaenoic acid (EPA) concentrations in plasma phospholipids. Consistent differences between summer and hibernation in bears and dormice were decreased ALA and EPA and marked increase of n-3 docosapentaenoic acid and a minor increase of docosahexaenoic acid in parallel with several hundred percent increase of the activity index of elongase ELOVL2 transforming C20-22 fatty acids. The highest LA supply was unexpectantly associated with the highest transformation of the n-3 fatty acids. Similar fatty acid patterns in two contrasting hibernating species indicates a link to the hibernation phenotype and requires further studies in relation to consciousness and metabolism.

## Introduction

Hibernation is an enigmatic physiological long-term state during winter when the animal’s metabolism, body temperature, heart rate and breathing rate drop to lower levels enabling animals to conserve energy and to survive through cold and harsh weather conditions when food is scarce (for reviews see [1–3]). In particular, the state of torpor (usually in animals < 5 kg body weight), corresponds to an active and substantial reduction of metabolic rate associated with a marked decrease of animal´s body temperature. In most hibernators, torpor is regularly interrupted by periodic interbout arousals (IBA), phases of high metabolism and euthermic body temperatures lasting only few hours [4, 5]. A different pattern is found in hibernating bears, which reduce their metabolic rate to ≈25% of basal rates, while lying dormant at constant and moderate hypothermia [6]. Under similar extreme conditions, humans would suffer from loss of lean body mass, heart failure, thrombosis, atherogenesis, azotaemia, and osteoporosis, but hibernators return to active state without organ injuries [7–13]. If the factors that trigger and initiate hibernation would be identified this could potentially have important implications for long-term lethargic conditions related to extensive surgery as organ transplantation.

The metabolic changes during hibernation imply that cellular membranes can maintain vital metabolism at extreme physiological temperatures and reduced metabolic rate [14–16]. Membrane function is highly dependent on the lipid composition, which changes prior to and during hibernation [17–19]. Seasonal changes of the intake of essential fatty acids, linoleic (LA, 18:2n-6) and alpha-linolenic (ALA, 18:3n-3) acids, and in the associated eicosanoid cascade have been reported among hibernators [20], including alpine marmots (*Marmota marmota*) [21] and brown bears (*Ursus arctos*) [22]. Investigations of skeletal muscle, white adipose tissue and plasma of hibernating bears have shown profound differences in both n-3 fatty acids and monounsaturated fatty acids [23]. In general, fatty acids are associated with hibernation performances, as evidenced by effects of increasing content of polyunsaturated fatty acids, notably of the n-6 series, in diets or in white adipose tissue stores on the propensity of overall energy savings during hibernation [24–30]. The n-3 fatty acids have been shown to increase in marmot heart and liver during winter, resembling the total lipid composition of brown adipose tissue with an increase of DPAn-3 and DHA at onset of hibernation. These are changes not seen in the white adipose tissue, except during spring [26].

Associations between low n-3 fatty acids and consciousness and sleep disturbances have been of interest in many studies in humans [31–41] and in animals [42]. However, the specific roles of omega fatty acids or the mechanisms by which lipids would act on consciousness remain unclear. For instance, a study investigating healthy British 7-9 years old school children reported that 40% of sleep problems were moderately but significantly associated to low circulating levels of docosahexaenoic acid (DHA, 22:6n-3) and lower ratio of DHA/arachidonic acid (AA, 20:4 n-6). In a subsample, actigraphy measures indicated better sleep at night and fewer wake episodes following DHA supplementation [31]. Given this knowledge, it is relevant to ask whether such processes also occur during hibernation, a state of lethargy that shares some metabolic similarities with consciousness and sleep including the conservation of energy [43]. It is still unclear how the hibernation in bears with its different pattern to the state of torpor and arousals in dormice are regulated [44–47]. The potential implications and regulatory roles of lipids, and notably of n-3 long-chain fatty acids, in the process of lethargy and sleep deprivation and hibernation have been discussed in relativly few investigations and remain to date unclear [26, 48–50]. In cell cultures of mice adipocytes from brown and white fat mass DPAn-3 was synthesized from labelled d5-ALA in both white and brown undifferentiateed adipose cells. It was more expressed in differentiated white adipose tissue than in brown cells, which showed a broader upregulation of different n-3 fatty acids [51]. This might be related to thermogenesis since supplementation by polyunsaturated fatty acids, like LA, ALA, and EPA, upregulated the of UCP2mRNA levels in white adipose cells [52].

In this context, the aim of this study was to investigate the seasonal changes of the n-3 fatty acid profile and the associated metabolism in two species contrasting in their hibernation behaviors, the free-ranging brown bear (*Ursus arctos*) and the garden dormouse (*Eliomys quercinus*). While bears hibernate at constant moderate hypothermia with body temperature ranging 32-34°C, garden dormice have a cyclic hibernation pattern of multiday bouts of deep torpor with body temperature down to 1-4°C interspaced by periodic arousal phases of euthermic temperatures (35-37°C). LA and ALA have been shown to influence physiological parameters as temperature regulation [23, 53], heart metabolism [54], signalling of nuclear factor kappa-light chain-enhancer of activated B cells (NFĸB), peroxisome proliferator-activated receptors (PPARs) including coactivator PGC-1α and anti-oxidant defence in adipose and liver tissues of dormice prior to hibernation [55, 56]. Frank et al. [57] have in a series of works shown that high ALA diet to squirrels increases the propensity to enter and remain in torpor and that high LA intake in the fall inhibited torpor and suggested that was associated to higher mortality in young squirrels [58]. A limitation of these studies is that only LA and ALA are studied, limiting the possibility to compare with our study. Changes in fatty acids, notably those of the n-3 family across different seasons including hibernation, is of increasing interest [19, 48]. Hence, we found it relevant to investigate if different proportions of LA and ALA influence the n-3 fatty acid profile during hibernation. This was performed in the small hibernator, the garden dormouse with a different hibernation pattern compared to the bears, but with the general purpose to find common denominators in fatty acid metabolism related to the hibernation phenotype.

## Results

### Bears

The concentration of palmitic acid (16:0) was 25% higher during winter, but longer saturated fatty acids showed significantly lower concentrations during winter compared to summer (Table 1). There was no gender difference except a slightly higher oleic acid (OA, 18:1n-9) concentration in the males (p = 0.01). Palmitoleic acid (16:1n-7) showed a trend to higher concentrations (p = 0.056) and nervonic acid (24:1n-9) was significantly (p<0.001) increased during hibernation. The eicosatrienoic acid (Mead acid, 20:3n-9) increased 1.4-fold and the ratio between AA and Mead acid, the T:T (triene/tetraene) ratio, increased nearly 4-fold (p<0.001). The phospholipid fatty acid profiles showed highly significant differences in most fatty acid concentrations of the n-6 and the n-3 series between hibernation and summer (Table 1), but LA did not change and its products, di-homo-γ-linolenic acid (DGLA, 20:3n-6) and AA increased and decreased, respectively. (See S1 Fig in S1 File for an overview of essential fatty acid transformations).

**Table 1:** Major plasma phospholipid fatty acids (mol%) in bears during summer and hibernation.

Fatty acid	Sommar	Winter	p-value
	N = 11	N = 11	
14:0 (Myristic)	0.2 (0.2-0.2)	0.1 (0.1-0.1)	<0.001
16:0 (Palmitic)	15.2 (14.3-16.3)	20.0 (19.8-20.6)	<0.001
18:0 (Stearic)	22.7 (22.2-23.0)	23.0 (22.0-23.4)	0.92
20:0 (Arachidic)	1.9 (1.8-1.9)	1.3 (1.2-1.5)	<0.001
22:0 (Behenic)	1.5 (1.4-1.6)	1.6 (1.3-1.8)	0.15
24:0 (Lignoceric)	1.3 (1.0-1.3)	0.9 (0.8-1.0)	<0.001
ΣSFA	43.0 (41.2-43.8)	46.8 (45.6-47.8)	<0.001
16:1 (Palmitoleic)	0.4 (0.4-0.5)	0.6 (0.4-0.8)	0.056
18:1 (Oleic)	17.5 (16.4-21.6)	18.3 (14.8-21.0)	0.45
ΣMUFA	21.2 (19.7-24.1)	23.9 (19.1-26.0)	0.41
24:1 n-9 (Nervonic)	2.4 (2.1-2.9)	4.2 (3.8-5.0)	<0.001
18:2 n- 6 (Linoleic)	11.9 (11.0-12.3)	11.3 (10.5-12.9)	0.95
20:3 n-6 (Dihomo-γ-linolenic)	1.0 (1.0-1.1)	1.9 (1.7-2.1)	<0.001
20:4 n-6 (Arachidonic)	15.3 (13.8-17.0)	9.3 (8.0-10.8)	<0.001
Σ n-6	28.3 (27.1-30.1)	23.6 (20.6-25.7)	<0.001
18:3 n-3 (α-Linolenic)	1.1 (0.8-1.3)	0.3 (0.2-0.4)	<0.001
20:5 n-3 (EPA)	2.4 (2.0-3.3)	0.4 (0.4-0.5)	<0.001
22:5 n-3 (DPA)	1.6 (1.4-2.0)	2.7 (2.4-3.1)	0.005
22:6 n-3 (DHA)	1.0 (0.4-1.2)	1.8 (1.6-2.6)	<0.001
Σ n-3	5.8 (5.6-6.6)	5.2 (5.0-6.3)	0.16
n-6/n-3	4.5 (4.3-5.1)	4.2 (3.6-4.7)	0.082
n-3 index	3.6 (2.9-3.7)	2.2 (2.0-2.9)	0.010
EDD index	4.9 (4.8-5.3)	5.0 (4.6-6.0)	0.92
20:3 n-9 (Mead)	0.7 (0.5-1.0)	1.9 (1.2-2.4)	<0.001
T:T ratio	0.0 (0.0-0.1)	0.2 (0.1-0.3)	<0.001

Data expressed as median and interquartile range (IQR). Wilcoxon´s test.

SFA, saturated fatty acids; MUFA, monounsaturated fatty acids; EPA, eicosapentaenoic acid; DPA, docosapentaenoic acid; DHA, docosahexaenoic acid; Omega-3(n-3)index, ΣEPADHA

EDD index, ΣEPA, DPA, DHA; T:T, trien:tetraen ratio (Mead acid/arachidonic acid).

**Fig 1:**
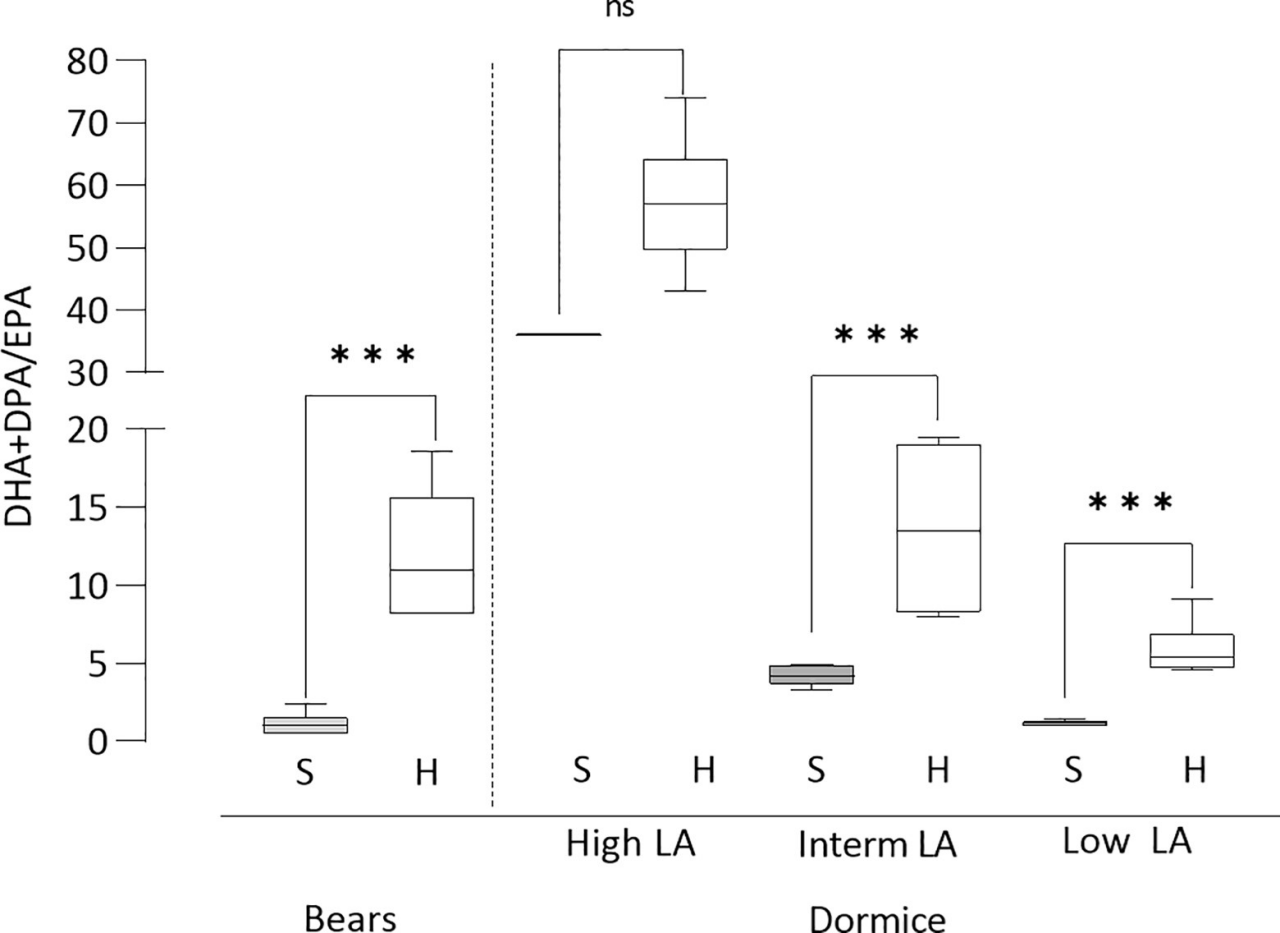
The ratio of DHA+DPA to EPA in plasma phospholipids in bears and dormice. Docosahexaenoic acid (DHA) and docosapentaenoic acid (DPA) to eicosapentaenoic acid (EPA) in bears and dormice with high, intermediate, or low linoleic acid (LA) content in diets. S, summer; H, hibernation. Box plots show median, 25^th^ and 75^th^ percentiles and whiskers show 10^th^ and 90^th^ percentiles.

The largest differences between seasons were observed in the n-3 fatty acids; ALA and EPA decreased, and docosapentaenoic acid (DPA, 22:5n-3) and DHA increased during hibernation. Changed balance between the n-6 and n-3 fatty acids was evident in the ratios between different fatty acids in the bears and in the dormice with low or intermediate LA intake (Table 2). The DPA/EPA and DHA/EPA ratios showed marked increases, without any changes in the total n-3 or the total n-3 long-chain polyunsaturated fatty acid (LCPUFA) concentrations (Table 1). Thus, it was mainly the relative order of the n-3LCPUFA which changed, illustrated by the 10-fold increase of the ratio DPA+DHA/EPA (Fig 1) and more than 3-fold increase of the ratio of the sum of EPA+DPA+DHA (EDD index) to ALA.

**Table 2:** Ratios of fatty acids in plasma phospholipids in bears and dormice during summer (S) and hibernation (H). The dormice are reported in groups related to different dietary – high, intermediate, or low – linoleic acid concentrations during summer (S) and torpor (T).

	Bears			Dormice								
				High LA			Intermediate LA			Low LA		
	S n = 11	H n = 11	p	S n = 7	T n = 6	p	S n = 8	T n = 4	p	S n = 7	T n = 6	p
LA/ALA	9.8 (8-16)	35 (28-56)	<0.001	121 (71-161)	247 (87-251)	0.063	23 (21-25)	36 (34-45)	0.007	9.5 (9-10)	17 (14-19)	0.003
AA/LA	1.2 (1.2-1.4)	0.83 (0.8-0.9)	<0.001	0.48 (0.3-0.7)	0.57 (0.6-0.)	0.67	0.36 (0.33-0.40)	0.48 (0.42-0.54)	0.027	0.31 (0.24-0.36)	0.46 (0.39-0.52)	0.015
AA/EPA	5.1 (4.1-11)	21 (16-30)	<0.001	174* (174-174)	145 (140-146)	0.13	12 (11-13)	21.5 (16-27)	0.007	2.8 (2.5-3.3)	8.3 (7.4-10)	0.003
AA/DHA	22 (13-34)	4.8 (3.9-6.6)	<0.001	5.5 (5.1-6.3)	2.9 (2.6-3.4)	0.003	3.3 (3.1-3.7)	2.2 (1.9-2.4)	0.007	2.9 (2.3-3.8)	1.9 (1.7-2.2)	0.007
DPA/EPA	0.7 (0.4-1.0)	6.0 (5.0-8.5)	<0.001	2.0* (2.0-2.0)	8.0 (7.0-9.0)	0.13	0.63 (0.58-0.71)	3.1 (2.0-4.4)	0.007	0.22 (0.20-0.26)	1.2 (0.94-1.4)	0.003
DHA/EPA	0.3 (0.1-0.5)	4.5 (3.2-8.7)	<0.001	34* (34-34)	50 (44-52)	0.13	3.6 (3.1-4.1)	10 (6.6-14)	0.007	0.94 (0.76-1.1)	4.0 (3.6-5.1)	0.003
DHA/DPA	0.4 (0.4-0.7)	0.6 (0.5-1.1)	0.028	14 (9-15.5)	6.1 (5.8-7.2)	0.003	5.2 (5.1-5.9)	3.3 (2.9-3.8)	0.006	3.9 (3.4-4.4)	3.1 (2.9-3.8)	0.086
EDD/ALA	4.8 (3.8-6.0)	16 (12-25)	<0.001	9.3* (6.7-15)	55 (20-62)	0.005	3.5 (3.0-4.3)	11 (10.5-14)	0.007	2.7 (1.4-3.1)	5.4 (5.1-7.6)	0.003
DHA+DPA/EPA	1.0 (0.46-1.5)	11 (8.2-16)	<0.001	36* (36-36)	57 (52-61)	0.13	4.2 (3.7-4.9)	13.5 (8.6-19)	0.007	1.2 (0.96-1.3)	5.4 (4.8-6.1)	0.003
EPA+DPA/DHA	6.5 (3.0-11)	1.9 (1.1-2.2)	<0.001	0.08* (0.07-0.11)	0.19 (0.16-0.19)	0.003	0.46 (0.42-0.52)	0.41 (0.38-0.45)	0.23	1.4 (1.1-1.6)	0.58 (0.54-0.60)	0.003

*During summer EPA was not measurable in 5/7 dormice reducing the number investigated. Median (IQR). Wilcoxon´s test for bears and Non-parametric ANOVA (Kruskall-Wallis) test for dormice. LA, linoleic acid; ALA, α-linolenic acid; AA, arachidonic acid; EPA, eicosapentaenoic acid; DHA, docosahexaenoic acid; DPA, docosapentaenoic acid; EDD, ΣEPA, DPA, DHA.

### Garden dormice

The fatty acid profiles showed no significant differences between late and early torpor and were thus combined representing torpor *vs* the summer active period (Table 3). There were neither difference in the fatty acid profiles between early and late IBA intervals, which hence were also combined into one group in the comparison between the periods (S1 Tables in S1 File). The three different diets regarding the LA proportions (see Methods) influenced the fatty acid profiles, and the groups were thus analyzed separately.

**Table 3:** Major plasma phospholipid fatty acids (mole%) in dormice during summer and torpor.

	Summer				Torpor			
	High LA	Intermediate	Low LA		High LA	Intermediate	Low LA	
	n = 7	n = 8	n = 7	p^A^	n = 6	n = 4	n = 6	p^A^
14:0 (Myristic)	0.10 (0.10-0.10)	0.05 (0.00-0.10)	0.00 (0.00-0.10)^§^	0.23	0.10 (0.10-0.10)	0.10 (0.10-0.10)	0.10 (0.10-0.10)	0.43
16:0 (Palmitic)	23 (22-23) ^#^	22 (21-22.5)^#^	22.5 (22-24) ^#^	0.25	26.5 (26-27)	26 (25-26)	26 (26-26)	0.13
18:0 (Stearic)	17(16-17) ^#^	17 (16.5-17) ^#^	16 (15-17) ^#^	0.15	10 (10-11)	11 (10-12)	11 (10-11)	0.86
20:0 (Arachidic)	0.20 (0.10-0.20)^a^	0.20 (0.10-0.20)^a,b^	0.10 (0.10-0.10)^b^	0.02	0.20 (0.10-0.20)^a^	0.10 (0.10-0.10)^a,b^	0.10 (0.10-0.10)^b^	0.017
22:0 (Behenic)	0.60 (0.60-0.60)^§^	0.60 (0.50-0.60)	0.50 (0.50-0.60)	0.26	0.65 (0.60-0.70)	0.65 (0.60-0.70)	0.60 (0.50-0.60)	0.34
24:0 (Lignoceric)	0.40 (0.40-0.50)	0.40 (0.40-0.45)	0.40 (0.40-0.50)	0.84	0.50 (0.50-0.50)	0.45 (0.40-0.55)	0.50 (0.40-0.50)	0.81
ΣSFA	40 (40-41) ^#^	40 (40-41)	40 (40-40)	0.32	38 (38-39)	37.5 (37-39)	38 (37-38.5)	0.32
16:1 n-7 (Palmitoleic)	0.20 (0.20-0.20) ^#^	0.20 (0.20-0.20) ^§^	0.20 (0.20-0.20) ^#^	0.55	0.45 (0.40-0.50)	0.45 (0.40-0.50)	0.40 (0.30-0.50)	0.58
18:1 n-9 (Oleic)	7.9 (7.8-8.4) ^#a^	9.6 (9.2-10) ^#a,b^	11 (10-11) ^#b^	<0.001	13 (12-13)^a^	14 (14-14)^a,b^	16 (15.5-16)^b^	0.002
24:1 n-9 (Nervonic)	0.90 (0.70-1.0) ^#a^	0.75 (0.70-0.85) ^#a,b^	0.60 (0.50-0.70) ^#b^	0.002	1.55 (1.4-1.6)	1.55 (1.5-1.7)	1.35 (1.2-1.5)	0.14
ΣMUFA	9.0 (8.7-9.7) ^#a^	11 (10-11) ^#a,b^	12 (11-12) ^#b^	<0.001	15 (14-15)^a^	16 (16-16)^a,b^	17.5 (17-18)^b^	0.003
18:2 n-6 (Linoleic)	32 (26-36) ^§^	31 (30-32) ^#^	28 (26.5-32) ^#^	0.61	25 (25-26)	25 (24-26)	23 (21-26)	0.67
20:3 n-6 (DGLA)	0.30 (0.30-0.40) ^#a^	0.50 (0.40-0.50) ^#b^	0.50 (0.50-0.70)^b^	0.002	0.85 (0.80-0.90)^a^	0.85 (0.65-1.00)^a^	0.60 (0.60-0.70)^a^	0.049
20:4 n-6 (AA)	15.5(11-17)^a^	11 (10.5-12)^a,b^	8.8 (7.6-9.5) ^§^^c^	<0.001	14.5 (14-15)^a^	12 (11-13) ^a,b^	11(10-11)^b^	0.003
Σ n-6	48 (45-48) ^#a^	43 (42-43.5) ^#a,b^	38 (36.5-40) ^#b^	<0.001	40.5 (40-41)^a^	38 (37-38) ^a,b^	35 (33-36)^b^	0.001
18:3 n-3 (ALA)	0.30 (0.20-0.40) ^§^^a^	1.35 (1.25-1.45) ^#a,b^	3.0 (2.7-3.5) ^#b^	<0.001	0.10 (0.10-0.30)^a^	0.70 (0.60-0.70) ^a,b^	1.4 (1.3-1.4)^b^	0.001
20:5 n-3 (EPA)	0.00 (0.00-0.00) ^#a^	0.95 (0.85-1.05) ^§^^a,b^	3.2 (2.3-3.9) ^#b^	<0.001	0.10 (0.10-0.10)^a^	0.60 (0.40-0.80) ^a,b^	1.35 (1.0-1.5)^b^	0.001
22:5 n-3 (DPA)	0.20 (0.20-0.20) ^#a^	0.60 (0.55-0.65) ^#b,c^	0.70 (0.50-0.90) ^#c^	<0.001	0.80 (0.70-0.90)^a^	1.7 (1.4-1.95)^b^	1.6 (1.4-1.8)^b^	0.005
22:6 n-3 (DHA)	2.9 (1.8-3.4) ^#^	3.35 (3.0-3.75) ^#^	3.0 (1.9-4.0) ^#^	0.41	4.95 (4.4-5.2)	5.55 (5.3-5.7)	5.1 (4.3-6.5)	0.47
Σ n-3	3.3 (2.3-4.1) ^#a^	6.25 (6.1-6.65) ^#a,b^	9.5 (8.6-11)^b^	<0.001	6.0 (5.4-6.3)^a^	8.45 (8.05-8.8) ^a,b^	9.6 (8.5-11)b	0.004
Omega 3 index	2.9 (1.8-3.5)^a^	4.2 (4.05-4.75) ^#a,b^	6.2 (4.4-7.5)^b^	0.001	5.05 (4.5-5.3)	6.1 (6.0-6.25)	6.45 (5.1-8.3)	0.12
EDD index	3.1 (2.0-3.7)^a^	4.75 (4.65-5.4) ^#b,c^	6.9 (4.9-8.4)^c^	<0.001	5.8 (5.3-6.2)^a^	7.85 (7.35-8.2)^a,b^	8.1 (7.1-10)a	0.018
20:3n-9 (Mead)	0.20(0.20-0.20)	0.20(0.20-0.20)	0.20(0.20-0.20)	0.61	0.20(0.20-0.20)	0.20(0.20-0.20)	0.20(0.20-0.20)	0.63
T:T ratio	0.01 (0.01-0.02)^a^	0.02 (0.02-0.02)^a,b^	0.03 (0.02-0.03)^b^	0.002	0.01 (0.01-0.01)^a^	0.02 (0.01-0.02)^a^	0.02 (0.01-0.02)^a^	0.029

Hibernation constituted early and late torpor combined. The dormice were grouped according to different concentrations of dietary linoleic acid, high, intermediate, and low. Median (IQR). Non-parametric ANOVA (Kruskall-Wallis) for summer and torpor, respectively, marked as p^A^, and significant differences between the dietary groups within each season labelled with letters a, b and c (Dunn’s test).^.^Significant p values between summer-torpor for each diet group

*p<0.001

^#^p<0.01

^§^p<0.05 (Wilcoxon). DGLA, dihomo-γ-linolenic acid; AA, arachidonic acid; ALA, α-linolenic acid; EPA, eicospentaenoic acid; DPA, docosapentaenoic acid; DHA, docosahexaenoic acid; Omega-3 index, ΣEPA,DHA; EDD index, ΣEPA,DPA,DHA; T:T ratio, Mead acid/Arachidonic acid.

The longer saturated fatty acids showed the same pattern in all diet groups with a rise in palmitic acid and decrease in stearic acid (18:0) during hibernation. All analyzed monounsaturated fatty acids increased during torpor. Generally, there was no difference between torpor and IBA in fatty acid concentrations (S1 Tables in S1 File). LA was not different between groups, and the decrease during hibernation was similar across all diets. DGLA increased, but not in the group with low LA intake. The dietary influence was largest for AA with low concentrations in summer and increase during torpor at low LA intake, but no changes were seen in the high and intermediate LA groups during torpor.

The influence of the diet was also seen in the n-3 series, where the expected increased concentrations of ALA with decreasing LA/increasing ALA intake was not seen, and in all dietary groups ALA decreased during torpor. EPA was very low, often not measurable during summer in the high LA group but decreased markedly during torpor in the other dietary groups. Contrary to those patterns, the 22C n-3 fatty acids, DPA and DHA, raised markedly during hibernation in all dietary groups, but DHA was the only n-3 fatty acid not differing between groups in summer and hibernation, respectively. The differences between the omega-3 index (ΣEPA, DHA) and the EDD index strongly supported that the main difference between the active summer period and hibernation was the increase of DPA (Table 3 and S1 Tables in S1 File). In the groups with high and intermediate LA intakes, the increase of DPA was 280-300% but in the low LA intake group only 130-200%, despite its higher ALA intake. Corresponding values for DHA was 70-124% and 70-80%, respectively. Generally, differences related to diet were most expressed in analyses of ratios of fatty acids (Table 2). The ratio DHA+DPA /EPA showed exceptional high values in the high LA dietary group during summer and hibernation compared to the other dietary groups, despite its lower ALA intake (Fig 1).

### Enzyme activity index in bears and dormice

The enzyme activities related to the fatty acid transformation were calculated as index from the ratio of the involved fatty acids (Table 4 and S1 Fig in S1 File). Stearoyl-CoA desaturase (SCD_18_, Δ9-desaturase) index did not change in bears but increased in the dormice during torpor and IBA with highest values in the low LA group, consistent with the increase of OA. Palmitoleic acid increased in all dietary groups in the dormice and SCD_16_ index showed a low but significant increase during torpor. FADS1 index, reflecting transformation of DGLA to AA, decreased in bears during hibernation, but was consistently high in the dormice, and increased slightly and significantly during hibernation in the low LA intake group, consistent with the small changes in the AA concentration. FADS2 index, reflecting the desaturase transforming LA and ALA in the first step to longer fatty acids, showed increased activity in both bears and dormice during hibernation.

**Table 4:** Enzyme indices illustrating putative activities of desaturases and elongases in bears and dormice.

	Bears			Dormice											
				High LA				Intermediate LA				Low LA			
Enzyme index	S n = 11	H n = 11	p	**S** **n = 7**	T n = 6	IBA n = 8	p	S n = 8	T n = 4	IBA n = 5	p	S n = 7	T n = 6	IBA n = 5	p
SCD16	0.03 (0.02-0.03)	0.03 (0.02-0.04)	0.77	0.01 (0.01-0.01)	0.02 (0.01-0.02)	0.01 (0.01-0.02)	0.003	0.01 (0.01-0.01)	0.02 (0.02-0.02)	0.01 (0.01-0.02)	0.002	0.01 (0.01-0.01)	0.02 (0.01-0.02)	0.01 (0.01-0.01)	0.003
SCD18	0.79 (0.70-0.93)	0.84 (0.64-0.93)	0.45	0.48 (0.45-0.52)	1.2 (1.1-1.3)	1.2 (1.1-1.3)	0.001	0.56 (0.53-0.61)	1.3 (1.1-1.4)	1.3 (1.2-1.3)	0.002	0.69 (0.63-0.71)	1.5 (1.4-1.5)	1.4 (1.35-1.6)	0.002
FADS1	22 (18-28)	4.7 (3.1-9.0)	<0.001	77 (56-87)	72 (70-73)	77 (69-81)	0.36	57 (53-59)	59 (54-64)	60 (57-61)	0.60	44 (38-48)	52 (50-55)	54 (50-59)	0.018
FADS2	0.09 (0.03-0.11)	0.17 (0.14-0.20)	<0.001	0.09 (0.05-0.13)	0.20 (0.17-0.21)	0.22 (0.20-0.25)	0.001	0.11 (0.09-0.13)	0.22 (0.21-0.23)	0.32 (0.25-0.34)	0.001	0.11 (0.06-0.14)	0.23 (0.16-0.31)	0.28 (0.25-0.29)	0.008
ELOVL2	0.67 (0.36-1.0)	6.0 (5.0-8.5)	<0.001	2.0 (2.0-2.0)	8.0 (7.0-9.0)	8.0 (7.0-8.0)	0.23	0.63 (0.58-0.71)	3.1 (2.0-4.4)	3.0 (2.2-3.50)	0.002	0.22 0.20-0.26)	1.2 (0.94-1.4)	1.7 (1.6-1.9)	0.002
ELOVL5	2.7 (2.2-3.3)	1.3 (1.0-2.0)	0.023	0.00 (0.00-0.00)	1.0 (0.33-1.0)	1.0 (1.0-1.0)	<0.001	0.69 (0.59-0.84)	0.97 (0.69-1.1)	0.71 (0.57-0.83)	0.48	1.2 (0.68-1.3)	0.90 (0.77-1.1)	0.79 (0.77-1.1)	0.91

Enzyme indices tentatively illustrating activities of desaturases and elongases in the major transformation steps of endogenous and essential fatty acids in bears and dormice during summer and hibernation. Data for bears during summer (S) and hibernation (H), and for dormice during summer (S), torpor (T) and interbout intervals (IBA) in relation to diets containing high, intermediate, and low content of linoleic acid (LA). Stearoyl-CoA desaturase transforming palmitoleic acid from palmitic acid (SCD_16_), and oleic acid from stearic acid (SCD_18_). Desaturases as arachidonic acid/dihomo-γ-linolenic acid (FADS1) and ratio dihomo-γ-linolenic acid/linoleic acid (FADS2). Elongases as ratio of docosapentaenoic acid/eicosapentaenoic acid (ELOVL2) and ratio of eicosapentaenoic acid/α-linolenic acid (ELOVL5). Median (IQR). Wilcoxon´s test used for bears and non-parametric ANOVA (Kruskal-Wallis) test used for dormice.

The most marked change was found in the elongase activity index transforming C20-22 fatty acids, the elongase 2 (ELOVL2). The activity index indicated an increase of 800% in the bears and 400-500% in the dormice, irrespective of diet. The ELOVL5 activity index (tentatively reflecting the transformation of C18-C20 fatty acids) decreased during hibernation in the bears but did not change in the dormice except in the high LA intake group, where an increase was seen from mainly not measurable values during summer (Table 4).

## Discussion

The results of the present study of two different models of hibernation, in bears and dormice, suggest that elevated plasma phospholipid n-3 DPA is associated with the hibernation phenotype. We report marked differences in plasma phospholipid concentrations of n-3 fatty acids during hibernation compared with the active summer period. These changes were consistently found in bears and dormice, despite their different hibernation patterns. DHA was not influenced by the different n-6 and n-3 fatty acid intake in the dormice, but the extent of influence on the n-3 LCPUFA differed unexpectantly since the most marked changes were seen in the group with highest LA intake (Tables 2–4). Arnold et al. [26] reported an increase in the proportion of n-6 phospholipids in a short pre-hibernation phase. This finding is supported by our observation that the most pronounced increase of DPA and DHA was observed in the dormice with the highest LA intake. Rice et al. [48] found an increase in early arousal of long-chain fatty acids, both n-6 and n-3, but didn´t analyzed DPAn-3. Although DHA and DPA concentrations were low, their significant increases in plasma phospholipids during hibernation and the balance, as expressed in their ratios to the substrate fatty acids, showed extensive changes with 10-fold increase in bears of the ratio DPA+DHA to EPA. An even more pronounced increase was observed in the dormice, especially in association with high LA intake. This contradicts the general view that high n-3 fatty acids should favor the n-3 fatty acid metabolism. The increases during hibernation of DPA and DHA were associated with several hundred percentage increase of ELOVL2 activity index. In Atlantic salmon high vegetable oil diet, e.g. high n-6 intake, stimulated ELOVL2 but not ELOVL5 expression [59]. Thus, our results suggest that there is a link between the shown beneficial effect of LA supply with the increase of n-3LCPUFA during hibernation. Our results, suggesting that n-3 DPA is involved in hibernation, may thus support and explain the previous observation of delay in hibernation with a high n-3 diet combined with low LA intake [54]. In our study the animals with lowest n-6/n-3 ratio had the lowest ELOVL2 activity index.

Physiologically our results suggest an upregulated transformation of ALA, which in the bears might be associated with the high intake of plants and berries during autumn [60]. This observation was further supported by the consistent increase of FADS2 index and the decreasing concentrations of ALA and EPA supporting the higher transformation to DPA. A strong influence by ALA intake on DPA synthesis has previously been shown [61, 62], more pronounced when mixed with LA, which also influenced prostaglandin metabolism [61]. Furthermore, increase of DPA was reported in plasma and white adipose tissue in brown bears [23] and garden dormice during hibernation, [54]. Our data corroborate a report of increased concentrations of ELOVL2 in the liver of hibernating bears [63]. Genome wide association studies (GWAS) have found associations in humans between the expression of ELOVL2 and sleep duration [64, 65], supporting a physiological link between hibernation and consciousness. In a study of Greenland Innuits GWAS also showed associations between ELOVL2 and age and DNA methylation [65].

The dietary impact related to different organ functions might demand special attention [66, 67]. It has been suggested that the heart would be sensitive to LA concentrations by interfering with SERCA2 activity [53, 54]. In rats, the heart is sensitive for essential fatty acid deficiency and together with the liver, it was the tissue taking up most of labelled LA, when it was supplied to essential fatty acid deficient animals [68]. Rat heart was shown to lack elongase-2mRNA, making it dependent on plasma supply for adequate levels of DPA and DHA [69].

Both the low and high LA diet in the dormice was associated with substantial influences on the fatty acid profile. It is well known that low LA diet shows a compensatory increase of OA, which was shown also in summer in the animals of this study. Unexpectedly, the high LA intake group showed the highest increase of OA during torpor (63% *vs* 45-47% in the other dietary groups). All dietary groups had similar increases of the enzyme index of SCD_18_, which transforms stearic acid to OA, and the difference between summer and torpor showed a similar pattern regardless of diet (Table 4). The absence of an increase of Mead acid in the dormice, even in the low LA group was unexpected and might be related to the different hibernation pattern with increase of metabolism during IBA, with euthermic body temperature and higher metabolism with higher respiratory quotient indicating less lipid oxidation [3–5]. The changes in Mead acid and T:T index in the bears is more difficult to explain, since they did not show a more expressed decrease in LA, which usually is the reason for such changes [70]. On the other hand, they reduce their metabolic rate on average to 25% of basal rate while lying dormant at constant moderate hypothermia [6] and constant high lipid metabolism with low respiratory quotient. The increase of Mead acid might be a compensation for the marked decrease of AA, which was not seen in the dormice.

The strong association observed between DPA and DHA and the hibernation status raises several questions. Previous reports about feeding dormice with high DHA diet prior to hibernation has been found to delay its onset but without further impact on the deepness and duration of the individuals’ torpor bouts during winter [54]. This effect was explained by the authors through the fact that dormice lowered n-3 LCPUFA, namely DHA, proportions via selective mobilization/oxidation of these lipids before entering into deep torpor, hence delaying hibernation. Similar results have been reported in tropical daily heterotherms (*Microcebus murinus*), which had few hours in torpor and difficulties to reach lower temperature on EPA and DHA supplementation [71]. High levels of n-6 fatty acids, including LA, have been suggested to be important for successful hibernation [8, 53, 55, 56, 72]. Recent studies also indicate that the n-6/n-3 ratio influences the adipose lipid metabolism during hibernation [55, 56]. Thus, it is of interest that the group of dormice with the highest LA intake and the lowest ALA intake, showed the most marked increase of the ratio between DPA+DHA to EPA as well as the ratio EDD/ALA, suggesting a very high turnover to the n-3 22C fatty acids. The influence of PUFA or the n-6/n-3 ratio differs between species [8], but our results suggest importance of LA for the LCPUFA increase during hibernation.

Several clinical studies have suggested that higher n-3 fatty acid intake induces a deeper sleep [31–33, 36, 38, 40, 42]. Although the DPA content is second to DHA in the brain n-3 fatty acids [73], it is seldom analyzed [34, 35, 74], but has been associated with sleep [41, 64]. In Innuits, some of whom at least in older times consumed very high amounts of DPA-rich seal meat, providing them with daily amount of 1.7–4.0 g DPA [73], sleep paralyses have been reported [75]. Our observation suggests that a putative role of DPA in induction and maintenance of hibernation as well as in prolonged periods of consciousness and sleeping behavior needs attention.

Our study cannot differ if the increased activity index of ELOVL2 was associated with increased expression and/or increased activity. Epigenetic mechanisms would be the dynamic process to explain these rapid changes [76, 77] and many long-chain n-3 fatty acids are known to influence gene expression [78], including DPA [79, 80]. Hibernation is characterized by differential expression of common genes, probably related to both transcriptional and post-transcriptional gene regulation and protein modification, which seem to be tissue-specific and especially involving enzymes in lipid metabolism [81–83]. DNA methylation has been related to ELOVL2 expression [65, 84], and can be influenced by many methyl donors in the diet [85], such as betain that increases 4-fold during hibernation in the bears [86]. It is also possible that lipid mediators of DPA and/or DHA are the active regulators [87, 88]. Changes in gene expression is a field for metabolic regulation both in humans and in experimental settings [89, 90]. As changes during hibernation have many similarities to quiescence as studied in stem cells, this should stimulate for further mechanistic studies [91].

Other mechanisms for the n-3 fatty acids influencing sleep have been suggested. Animal studies have shown influence of n-3 fatty acids on the pineal gland and the melatonin production, but with diverging results [92, 93]. The ratio between n-6 and n-3 fatty acids has been suggested to influence sleep by the prostanoid metabolism [94] and melatonin release by activating lipoxygenases [55, 56, 93, 95]. Thus, lipidomics need to be further studied in relation to sleep and hibernation.

Some limitations of the study must be pointed out. The bears were all young adults, and they were only investigated once in summer and winter. The dietary grouping of the dormice led to small groups and single animals could not be investigated in different phases. Thus, any differences between early and late torpor, as well as early and late IBA could not be disclosed. However, as the sub-analyses in dormice showed consistent results regarding the n-3 fatty acid pattern with those observed in the bears, the results were robust despite these limitations. The present study implies that further investigations on potential mechanisms linking n-3 fatty acids to hibernation as well as sleeping behaviors are warranted.

## Material and methods

### Animals

**Bears.** Blood samples were taken from 11 free-ranging sub-adult 2-to 3-yr-old Eurasian brown bears equipped with a Global Positioning System (GPS) collar in Dalarna and Gävleborg´s Counties, Sweden during 2012-2014. Bears were captured during hibernation (February-March) with median (IQR) body weight 48 (29-54) kg and again during the summer active period (June), median (IQR) body weight 52.5 (41.5-61) kg. Details on sampling procedures have been presented elsewhere [96].

#### Garden dormice

In total 56 garden dormice obtained from a breeding colony kept at the Research Institute of Wildlife Ecology (Vienna, Austria) were included in these experiments. The dormice presented in this study were part of a large experiment conducted over 3 consecutive years where animals had to be sacrificed at different time-points during hibernation (torpor and euthermic) to assess to various tissues, including the heart and other organs of interest. Repeated blood samplings or tissue biopsy were not possible within the study design [55, 56, 97].

Animals were housed singly in cages (60 × 40 × 40 cm), each equipped with one nest, bedding, and nesting material, as previously reported [55]. Dormice (n = 22) were kept under natural fluctuations of ambient temperature (summer) with median (IQR) body weight 118 (111-125) g. In the photoperiod during their pre-hibernation fattening (September), until the hibernation period (October to January), 34 animals were housed with constant darkness, without food and water individually in standard laboratory cages (36 × 20 × 14 cm), each provided with a customized nest and bedding material, and kept at 4°C in ventilated cooling units (Liebherr GKv 5730, Germany). Their median (IQR) body weight was 137 (125-157) g.

The dormice were divided into three groups with different proportions of LA and ALA in agreement with previous studies showing influences of proportions of n-6 and n-3 fatty acids on hibernation, but with unclear conclusions, indicating need of further studies [26, 48, 54]. Each group received a tailored proportion of LA, low (19%), intermediate (36%) and high (53%) concentrations of the fatty acid. The ALA concentration differed in opposite direction, being 32%, 17% and 1.35%, respectively. The full dietary lipid composition (S2 Table in S1 File) has previously been published [56]. Each specific diet was provided to the dormice for two weeks during the summer (August) or during their pre-hibernation period (September) as previously described [55, 56, 98, 99]. Before hibernation, the dormice were surgically implanted with small temperature transmitters (TA-F10, 1.1cc, 1.6g, accuracy: 0.15°C; Data Sciences International, St Paul, MN, United States) to monitor the individual’s hibernating pattern during winter [54, 97]. All animals were sacrificed by decapitation during summer after the feeding treatments, and at different timepoints within the torpor-arousal cycle during hibernation in winter. Specifically, blood samples were obtained during summer, and winter hibernation in early (1-2 days) and late (9-10 days) torpor as well as in early (1-2 h) and late (3-5 h) IBA. During hibernation, torpid animals were sacrificed by immediate decapitation, whereas summer and winter euthermic individuals were first euthanized by incremental exposure to carbon dioxide (CO_2_) followed by decapitation, as previously described [97]. Fresh blood was collected immediately after decapitation in lithium heparinized tubes (LiHep Micro-tube, 1.3. ml, Sarstedt, Germany) via the trunk of the animals. Blood samples were centrifuged at 1000 g and plasma stored at -80°C until transferred to laboratory for analyses of fatty acids.

#### Ethics approvals

The field studies of the bears did not involve endangered or protected species. All animal handling and sampling was carried out under approval of the Swedish Ethical Committee on Animal Research (C212/9 and C268/12), Uppsala, Sweden and were following Swedish laws and regulations.

All procedures regarding dormice experiments were approved by the Ethics and Animal Welfare Committee of the University of Veterinary Medicine, Vienna in accordance with the University’s guidelines for Good Scientific Practice and authorized by the Austrian Federal Ministry of Education, Science and Research (ref BMWF – 68.205/0137-WF/V/3b/2014) in accordance with current legislation.

### Phospholipid fatty acid analyses

Plasma phospholipid fatty acids were studied since those analyses reflect relatively stable status and composition of membranes over weeks, while free fatty acids and triglycerides reflect the status in relation to sampling and feeding. In order to compare summer and winter with different feeding we made this preference, further supported by the fact that most metabolism is related to the activity in the membranes, where the phospholipids are the major influencers on the activity of different proteins (for review see [19, 100])

Plasma lipids were extracted using liquid-liquid extraction. Sample (200 μL) was added to a mixture of freshly made acidified methanol (80 μL 5M hydrochloric acid in 1 mL methanol). Chloroform was added and the sample was sonicated in water bath for 10 minutes before thoroughly mixed and centrifuged (1000g, 10 min). Chloroform phase was saved and evaporated under nitrogen (37°C).

Lipid separation was carried out on NH_2_-SPE columns (Sep-Pak Aminopropyl (NH_2_) 1 cc Vac Cartridge, 100 mg, WAT023610, Waters, Milford, MA, USA) preconditioned with hexane (1 mL twice). Sample was dissolved in 400 μL chloroform doped with 1 drop of BHT (butylated hydroxytoluene in methanol 5 mg/mL) and thoroughly mixed. The suspension was applicated on to the SPE-column, which was washed twice to eluate neutral and free fatty acids; first with chloroform:2-propanol (2:1 v/v) (1mL) and second with acetic acid (2%) in diethyl ether (1mL). Finally, phospholipids were washed out using methanol (1 mL) and the eluate evaporated under nitrogen (37°C). Phospholipids were dissolved in 2mL acidified methanol (3M hydrochloric acid in methanol) and heated (100°C, 1 hour) resulting in hydrolysis and methylation of fatty acids. After cooling ultra-filtrated water (800 μL) was carefully added. The product was extracted using liquid-liquid extraction with n-hexane (3 mL). Solution was mixed and centrifuged (1000g, 5 min), and the n-hexane phase was saved and evaporated under nitrogen (37°C).

The methylated fatty acids were resolved in heptane (100 μL). Analysis was carried out on a GC connected to a flame ionization detector (GC-FID HP6890, Agilent Technologies, Santa Clara, CA, USA) with cool on-column injector. The GC-column had polysiloxane as stationary phase (DB225, 30 m * 0,320 mm * 0,25 μm, 123-2232, Agilent Technologies, Santa Clara, CA, USA) and helium was used as carrier gas. The final ionization was carried out in a gas-mixture of helium (carrier), hydrogen (combustor), air/oxygen (oxidant) and additional nitrogen (make-up gas).

The fatty acids were identified by retention times in a reference chromatogram and the proportions are presented as molar % of total area of identified fatty acids. Non-specified fatty acids in the tables constituted 0-2% in the analyses from the bears and 0-1% in the analyses from the dormice.

Enzyme expression or activities were not analyzed but activity index was expressed as enzyme index, calculated from the ratios between the involved fatty acids, which has been accepted as a tentative measure for the activity [101–104].

### Statistical analyses

Data are expressed as median and (10^th^ or 25^th^) to (75^th^ or 90^th^) percentile, percentage or median and interquartile range (IQR) as appropriate. Fatty acids are expressed as molar % of analyzed fatty acids. Statistical significance was set at the level of P <0.05. Comparisons between two groups and more were assessed with the non-parametric Wilcoxon´s test or Kruskal-Wallis’s test for continuous variables and Chi-square test for nominal variables. All statistical analyses were performed using statistical software Stata 17.0 (Stata Corporation, College Station, TX, USA).

## Supporting information

S1 File This file contains all the supporting tables and figure. (DOCX)
