# Supplementary material for: Elevated plasma phospholipid n-3 docosapentaenoic acid concentrations during hibernation
Source: PLoS One. 2023 Jun 9;18(6):e0285782. doi: 10.1371/journal.pone.0285782 (PMC10256182; doi:10.1371/journal.pone.0285782)
Supplement: S1 File — (DOCX) [file pone.0285782.s001.docx]

**Supporting information**

**S1 Fig. Major transformation steps of fatty acids and the enzymes involved.**

**S1 Tables. The plasma phospholipid fatty acid concentrations in dormice with different linoleic acid dietary content**. A) high, B) intermediate, C) low, during summer, torpor and interbout arousals.

**S2 Table. Fatty acid composition of low, intermediate and high linoleic acid diets.** Fed to garden dormice for at least two weeks during their pre-hibernation fattening phase (from ref 63).

Linoleic acid α-Linolenic acid Oleic acid

18:2n-6 (LA) 18:3n-3 (ALA) 18:1n-9 (OA)

**Omega-6 (n-6) Omega-3 (n-3) Endogenous (n-9)**

Arachidonic acid Eicosapentaenoic acid Mead acid

20:4n-6 (AA) 20:5n-3 (EPA) 20:3n-9

Adrenic acid Docosapentaenoic acid

22:4n-6 22:5n-3 (DPA)

Osbond acid Docosahexaenoic acid

22:5n-6 22:6n-3 (DHA)

FADS2

ELOVL5

FADS1

FADS2,

ELOVL5

FADS1

(ELOVL5)

ELOVL2

ELOVL2

Tetracosaenoic acid Tetracosapentaenoic acid

24:4n-6 24:5n-3

FADS2

β-oxidation

**Essential fatty acids**

**S1 Fig. Major transformation steps of fatty acids and the enzymes involved.**

**S1 Table A.** The phospholipid fatty acid concentrations in dormice with **high** dietary linoleic

acid during summer and hibernation. Early and late torpor were combined, and early

and late interbout arousals (IBA). Median (IQR).Non-parametric ANOVA.

|  | **Summer** | **Torpor** | **IBA** | **p-value** |
| --- | --- | --- | --- | --- |
| Fatty acid. | **N=7** | **N=6** | **N=8** |  |
| 14:0 (Myristic) | 0.1 (0.1-0.1) | 0.1 (0.1-0.1) | 0.1 (0.1-0.1) | 0.65 |
| 16:0 (Palmitic) | 23 (22-23) | 26 (26-27) | 26 (25-26) | <0.001 |
| 18:0 (Stearic) | 17 (16-17) | 10 (10-11) | 11 (10-11.5) | 0.001 |
| 20:0 (Arachidic) | 0.2 (0.1-0.2) | 0.2 (0.1-0.2) | 0.2 (0.1-0.2) | 0.95 |
| 22:0 (Behenic) | 0.6 (0.6-0.6) | 0.6 (0.6-0.7) | 0.6 (0.6-0.7) | 0.11 |
| 24:0 (Lignoceric) | 0.4 (0.4-0.5) | 0.5 (0.5-0.5) | 0.5 (0.4-0.6) | 0.44 |
| 16:1 n-7 (Palmitoleic) | 0.2 (0.2-0.2) | 0.5 (0.4-0.5) | 0.3 (0.3-0.4) | <0.001 |
| 18:1 n-9 (Oleic) | 7.9 (7.8-8.4) | 13 (12-13) | 13 (12-14) | 0.001 |
| 24:1 n-9 (Nervonic) | 0.9 (0.7-1.0) | 1.6 (1.4-1.6) | 1.6 (1.4-1.6) | 0.001 |
| 20:3 n-9 (Mead) | 0.2 (0.2-0.2) | 0.2 (0.2-0.2) | 0.2 (0.2-0.2) | 0.98 |
| Mead/Arachidonic | 0.02 (0.02-0.02) | 0.02 (0.02-0.03) | 0.02 (0.02-0.02) | 0.018 |
| 18:2 n-6 (Linoleic) | 32 (26-36) | 25 (25-26) | 25 (23-26) | 0.011 |
| 20:3 n-6 (Dihomo-γ-linolenic) | 0.3 (0.3-0.4) | 0.9 (0.8-0.9) | 0.6 (0.6-0.8) | <0.001 |
| 20:4 n-6 (Arachidonic) | 15.5 (11-17) | 14 (14-15) | 16 (14-16) | 0.36 |
| 18:3 n-3 (α-Linolenic) | 0.3 (0.2-0.4) | 0.1 (0.1-0.3) | 0.1 (0.1-0.1) | 0.005 |
| 20:5 n-3 (EPA) | 0.0 (0.0-0.0) | 0.1 (0.1-0.1) | 0.1 (0.1-0.1) | <0.001 |
| 22:5 n-3 (DPA) | 0.2 (0.2-0.2) | 0.8 (0.7-0.9) | 0.8 (0.7-0.8) | <0.001 |
| 22:6 n-3 (DHA) | 2.9 (1.8-3.4) | 4.9 (4.4-5.2) | 5.5 (5.0-5.9) | 0.001 |
| ΣSFA | 40 (40-41) | 38 (38-39) | 38 (37.5-38.5) | 0.003 |
| ΣMUFA | 9.0 (8.7-9.7) | 15 (14-15) | 15 (14-15) | 0.001 |
| Σn-6 | 48 (45-48) | 40.5 (40-41) | 41 (40-41) | 0.001 |
| Σn-3 | 3.3 (2.3-4.1) | 6.0 (5.4-6.3) | 6.5 (5.9-7.0) | 0.001 |
| Omega 3 index | 2.9 (1.8-3.5) | 4.2 (4.1-4.75) | 6.2 (4.4-7.5) | 0.001 |
| EDD index | 3.1 (2.0-3.7) | 4.75 (4.7-5.4) | 6.9 (4.9-8.4) | <0.001 |
| Σn-6/Σn-3 | 14 (11-21) | 6.7 (6.3-7.1) | 3.9 (3.2-4.7) | <0.001 |
| 18:1/18:2 | 0.25 (0.2-0.3) | 0.5 (0.5-0.5) | 0.5 (0.5-0.6) | 0.001 |

**S1 Table B**. The phospholipid fatty acid concentrations in dormice with **intermediate**

dietary linoleic acid during summer and hibernation. Early and late torpor were

combined, and early and late interbout arousals (IBA). Median (IQR). Non-

parametric ANOVA.

|  | **Summer** | **Torpor** | **IBA** | **p-value** |
| --- | --- | --- | --- | --- |
| Fatty acid. | **N=8** | **N=4** | **N=5** |  |
| 14:0 (Myristic) | 0.1 (0.0-0.1) | 0.1 (0.1-0.1) | 0.1 (0.1-0.1) | 0.063 |
| 16:0 (Palmitic) | 22 (21-22.5) | 26 (24-26) | 26 (25.5-26.5) | 0.003 |
| 18:0 (Stearic) | 17 (16.5-17) | 11 (10-12) | 11 (11-11.5) | 0.002 |
| 20:0 (Arachidic) | 0.2 (0.1-0.2) | 0.1 (0.1-0.1) | 0.2 (0.2-0.2) | 0.025 |
| 22:0 (Behenic) | 0.6 (0.5-0.6) | 0.6 (0.6-0.7) | 0.5 (0.5-0.6) | 0.18 |
| 24:0 (Lignoceric) | 0.4 (0.4-0.5) | 0.5 (0.4-0.6) | 0.5 (0.5-0.5) | 0.30 |
| 16:1 n-7 (Palmitoleic) | 0.2 (0.2-0.2) | 0.5 (0.4-0.5) | 0.3 (0.3-0.4) | <0.001 |
| 18:1 n-9 (Oleic) | 9.6 (9.2-10.0) | 14 (14-14) | 14.5 (14-15) | 0.002 |
| 24:1 n-9 (Nervonic) | 0.8 (0.7-0.9) | 1.6 (1.5-1.7) | 1.6 (1.3-1.6) | 0.002 |
| 20:3 n-9 (Mead) | 0.2 (0.2-0.2) | 0.2 (0.2-0.2) | 0.2 (0.2-0.2) | 0.57 |
| Mead/Arachidonic | 0.02 (0.02-0.02) | 0.02 (0.02-0.02) | 0.02 (0.02-0.02) | 0.60 |
| 18:2 n-6 (Linoleic) | 31 (30-32) | 25 (24-26) | 23 (22-24) | 0.002 |
| 20:3 n-6 (Dihomo-γ-linolenic) | 0.5 (0.4-0.5) | 0.8 (0.6-1.0) | 0.7 (0.6-0.7) | 0.005 |
| 20:4 n-6 (Arachidonic) | 11 (10.5-12) | 12 (11-13) | 12 (11-12) | 0.60 |
| 18:3 n-3 (α-Linolenic) | 1.4 (1.3-1.4) | 0.7 (0.6-0.7) | 0.7 (0.7-0.7) | 0.002 |
| 20:5 w3n-(EPA) | 0.9 (0.9-1.1) | 0.6 (0.4-0.8) | 0.5 (0.4-0.5) | 0.004 |
| 22:5 n-3 (DPA) | 0.6 (0.6-0.6) | 1.7 (1.4-1.9) | 1.3 (1.2-1.4) | 0.002 |
| 22:6 n-3 (DHA) | 3.3 (3.0-3.8) | 5.5 (5.3-5.7) | 7.4 (6.0-7.5) | 0.001 |
| ΣSFA | 40 (39-41) | 37.5 (37-39) | 39 (39-39) | 0.013 |
| ΣMUFA | 11 (10-11) | 16 (16-16) | 16 (16-17) | 0.002 |
| Σn-6 | 43 (42-43.5) | 38 (37-38) | 35 (34-37) | 0.001 |
| Σn-3 | 6.3 (6.0-6.7) | 8.4 (8.0-8.8) | 9.8 (8.7-10.0) | 0.002 |
| Omega 3 index | 5.1 (4.5-5.3) | 6.1 (6.0-6.3) | 6.5 (5.1-8.3) | 0.12 |
| EDD index | 5.8 (5.3-6.2) | 7.9 (7.4-8.2) | 8.1 (7.1-10) | 0.018 |
| Σn-6/Σn-3 | 6.7 (6.2-7.6) | 4.4 (4.2-4.5) | 3.5 (2.9-4.2) | 0.003 |
| 18:1/18:2 | 0.3 (0.3-0.3) | 0.6 (0.55-0.6) | 0.6 (0.6-0.6) | 0.001 |

**S1Table C.** The phospholipid fatty acid concentrations in dormice with **low** dietary

linoleic acid during summer and hibernation. Early and late torpor were combined,

and early and late interbout arousals (IBA). Median (IQR). Non-parametric

ANOVA.

|  | **Summer** | **Torpor** | **IBA** | **p-value** |
| --- | --- | --- | --- | --- |
| Fatty acid | **N=7** | **N=6** | **N=5** |  |
| 14:0 (Myristic) | 0.0 (0.0-0.1) | 0.1 (0.1-0.1) | 0.1 (0.1-0.1) | 0.022 |
| 16:0 (Palmitic) | 23 (22-24) | 26 (26-26) | 27 (27-28) | 0.002 |
| 18:0 (Stearic) | 16 (15-17) | 11 (10-11) | 11 (9.8-11) | 0.002 |
| 20:0 (Arachidic) | 0.1 (0.1-0.1) | 0.1 (0.1-0.1) | 0.1 (0.1-0.1) | 0.27 |
| 22:0 (Behenic) | 0.5 (0.5-0.6) | 0.6 (0.5-0.6) | 0.6 (0.6-0.7) | 0.23 |
| 24:0 (Lignoceric) | 0.4 (0.4-0.5) | 0.5 (0.4-0.5) | 0.4 (0.4-0.6) | 0.60 |
| 16:1 n-7 (Palmitoleic) | 0.2 (0.2-0.2) | 0.4 (0.3-0.5) | 0.3 (0.3-0.3) | 0.002 |
| 18:1 n-9 (Oleic) | 11 (10-11) | 16 (15.5-16) | 16 (15-16) | 0.002 |
| 24:1 n-9(Nervonic) | 0.6 (0.5-0.7) | 1.4 (1.2-1.5) | 1.3 (1.3-1.4) | 0.002 |
| 20:3 n-9 (Mead) | 0.2 (0.2-0.2) | 0.2 (0.2-0.2) | 0.2 (0.2-0.2) | 0.37 |
| Mead/Arachidonic | 0.01 (0.01-0.02) | 0.01 (0.01-0.01) | 0.01 (0.01-0.01) | 0.36 |
| 18:2 n-6 (Linoleic) | 28 (26.5-32) | 23 (21-26) | 21 (20-22) | 0.005 |
| 20:3 n-6 (Dihomo-γ-linolenic) | 0.5 (0.5-0.7) | 0.6 (0.6-0.7) | 0.7 (0.5-0.7) | 0.30 |
| 20:4 n-6 (Arachidonic) | 8.8 (7.6-9.5) | 10 (10-11) | 10 (10-12) | 0.018 |
| 18:3 n-3 (α-Linolenic) | 3.0 (2.7-3.5) | 1.4 (1.3-1.4) | 1.3 (1.2-1.4) | 0.001 |
| 20:5 n-3 (EPA) | 3.2 (2.3-3.9) | 1.4 (1.0-1.5) | 1.1 (1.0-1.3) | 0.002 |
| 22:5 n-3 (DPA) | 0.7 (0.5-0.9) | 1.6 (1.4-1.8) | 2.1 (1.7-2.1) | 0.001 |
| 22:6 n-3 (DHA) | 3.0 (1.9-4.0) | 5.1 (4.3-6.5) | 5.5 (5.4-6.3) | 0.010 |
| ΣSFA | 40 (39.5-40) | 38 (37-38.5) | 39 (39-39) | 0.027 |
| ΣMUFA | 12 (11-12) | 17.5 (17-18) | 17 (17-18) | 0.002 |
| Σn-6 | 38 (36.5-40) | 34.5 (33-36) | 33 (33-33.5) | 0.003 |
| Σn-3 | 9.5 (8.6-11) | 9.6 (8.5-11) | 10 (9.4-11) | 0.93 |
| Omega 3 index | 5.6 (5.1-6.0) | 7.8 (6.4-8.0) | 7.1 (6.7-7.3) | 0.014 |
| EDD index | 6.4 (5.8-6.8) | 9.1 (8.1-9.2) | 9.0 (8.2-9.7) | 0.005 |
| Σn-6/Σn-3 | 6.3 (5.7-6.8) | 3.5 (3.4-4.2) | 3.2(3.0-3.5) | 0.002 |
| 18:1/18:2 | 0.4 (0.3-0.4) | 0.7 (0.6-0.8) | 0.7 (0.7-0.8) | 0.002 |

**S2 Table.** **Fatty acid composition of low, intermediate and high linoleic acid diets.** Fed to garden dormice for at least two weeks during their pre-hibernation fattening phase. Fatty acid proportions are expressed as % of total fatty acids. Values are means from four analyses per diet. ‘PUFA’ refers to polyunsaturated fatty acids, ‘MUFA’ to monounsaturated fatty acids, ‘SFA’ to saturated fatty acids, ‘∑ n-6’ to the sum of n-6 PUFA, ‘∑ n-3’ to the sum of n-3 PUFA, ‘n-6/n-3’ to the ratio between the sum of n-6 PUFA and the sum of n-3 PUFA, and ‘LA/ALA’ to the ratio between linoleic acid (LA 18:2 n-6) and linolenic acid (ALA 18:3 n-3). Data were previously published in Logan et al. 2020 (Table 1)(ref 56).

| **Fatty acid** | **LOW** | **INT** | **HIGH** |
| --- | --- | --- | --- |
|  |  |  |  |
| C14:0 | 0.62 | 0.62 | 0.61 |
| C15:0 | 0.08 | 0.08 | 0.08 |
| C16:0 | 13.55 | 13.95 | 14.29 |
| C16:1 (n-7) | 1.55 | 1.60 | 1.60 |
| C17:0 | 0.18 | 0.18 | 0.16 |
| C18:0 | 6.26 | 5.64 | 5.00 |
| C18:1 (n-9) | 25.94 | 24.61 | 23.25 |
| C18:2 (n-6) | 19.28 | 35.55 | 52.95 |
| C18:3 (n-3) | 31.92 | 17.16 | 1.35 |
| C20:4 (n-6) | 0.34 | 0.33 | 0.35 |
| C20:5 (n-3) | 0.05 | 0.09 | 0.09 |
| C22:5 (n-3) | 0.08 | 0.06 | 0.09 |
| C22:6 (n-3) | 0.15 | 0.15 | 0.18 |
| PUFA | 51.82 | 53.34 | 55.01 |
| MUFA | 27.49 | 26.20 | 24.86 |
| SFA | 20.69 | 20.46 | 20.14 |
| ∑ n-6 | 19.62 | 35.87 | 53.29 |
| ∑ n-3 | 32.20 | 17.47 | 1.71 |
| n-6/n-3 | 0.61 | 2.06 | 31.50 |
| LA/ALA | 0.61 | 2.08 | 41.22 |
